# Development of a Noninvasive Blood Glucose Monitoring System Prototype: Pilot Study

**DOI:** 10.2196/38664

**Published:** 2022-08-26

**Authors:** Maria Valero, Priyanka Pola, Oluwaseyi Falaiye, Katherine H Ingram, Liang Zhao, Hossain Shahriar, Sheikh Iqbal Ahamed

**Affiliations:** 1 Department of Information Technology Kennesaw State University Marietta, GA United States; 2 Department of Software Engineering and Game Development Kennesaw State University Marietta, GA United States; 3 Department of Exercise Science and Sport Management Kennesaw State University Kennesaw, GA United States; 4 Department of Computer Science Marquette University Milwaukee, WI United States

**Keywords:** diabetes, deep learning, machine learning, glucose concentration, noninvasive monitoring, optical sensors, glucose monitoring

## Abstract

**Background:**

Diabetes mellitus is a severe disease characterized by high blood glucose levels resulting from dysregulation of the hormone insulin. Diabetes is managed through physical activity and dietary modification and requires careful monitoring of blood glucose concentration. Blood glucose concentration is typically monitored throughout the day by analyzing a sample of blood drawn from a finger prick using a commercially available glucometer. However, this process is invasive and painful, and leads to a risk of infection. Therefore, there is an urgent need for noninvasive, inexpensive, novel platforms for continuous blood sugar monitoring.

**Objective:**

Our study aimed to describe a pilot test to test the accuracy of a noninvasive glucose monitoring prototype that uses laser technology based on near-infrared spectroscopy.

**Methods:**

Our system is based on Raspberry Pi, a portable camera (Raspberry Pi camera), and a visible light laser. The Raspberry Pi camera captures a set of images when a visible light laser passes through skin tissue. The glucose concentration is estimated by an artificial neural network model using the absorption and scattering of light in the skin tissue. This prototype was developed using TensorFlow, Keras, and Python code. A pilot study was run with 8 volunteers that used the prototype on their fingers and ears. Blood glucose values obtained by the prototype were compared with commercially available glucometers to estimate accuracy.

**Results:**

When using images from the finger, the accuracy of the prototype is 79%. Taken from the ear, the accuracy is attenuated to 62%. Though the current data set is limited, these results are encouraging. However, three main limitations need to be addressed in future studies of the prototype: (1) increase the size of the database to improve the robustness of the artificial neural network model; (2) analyze the impact of external factors such as skin color, skin thickness, and ambient temperature in the current prototype; and (3) improve the prototype enclosure to make it suitable for easy finger and ear placement.

**Conclusions:**

Our pilot study demonstrates that blood glucose concentration can be estimated using a small hardware prototype that uses infrared images of human tissue. Although more studies need to be conducted to overcome limitations, this pilot study shows that an affordable device can be used to avoid the use of blood and multiple finger pricks for blood glucose monitoring in the diabetic population.

## Introduction

### Background

Diabetes affects approximately one out of every 10 people in the United States [1]. Its prevalence has increased from 23.4 million Americans in 2015 to 30.3 million in 2021 and continues to rise at an alarming rate [2].

Successful management of diabetes involves monitoring blood glucose levels multiple times per day. The standard method for monitoring blood glucose concentration is through the use of a glucometer [3]. This device determines glucose concentration from a droplet of blood obtained from a finger prick or a laboratory blood draw. Taking repeated finger pricks over the course of a day is painful and creates a risk of infection at the collection site [4]. Therefore, noninvasive methods are an attractive alternative, however, those that are available today have several limitations.

**Figure 1:**
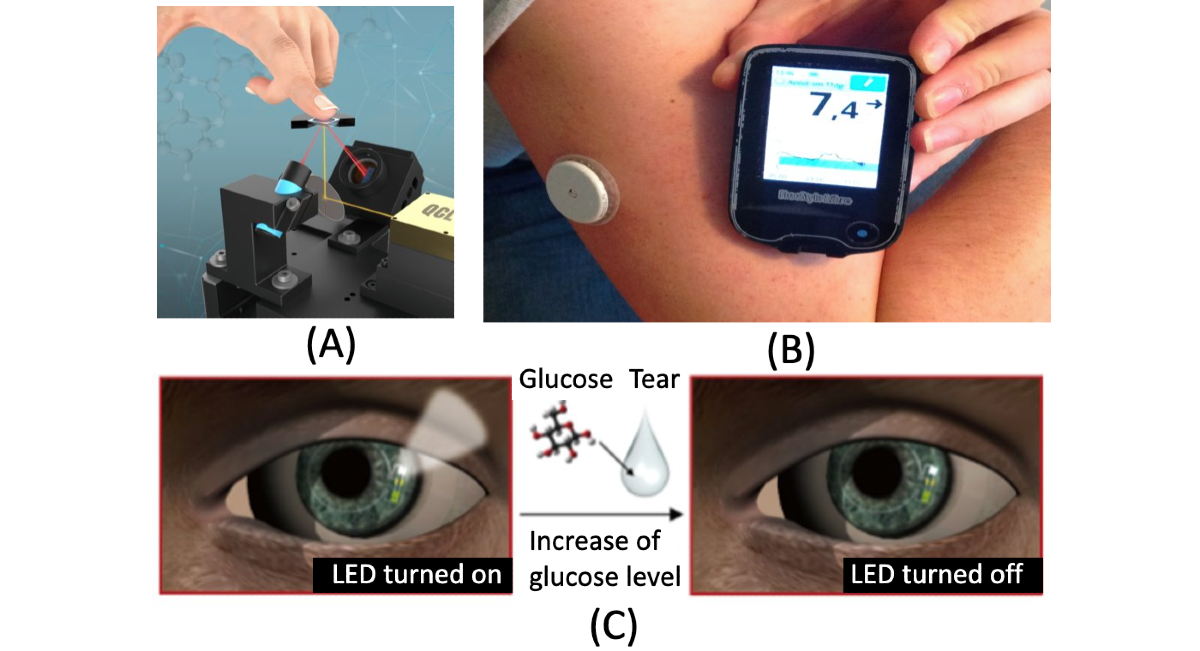
Examples of (A) NIO-GM (adapted from Lubinski et al [6]), (B) MI-GM (adapted from Sjö [7], published under Creative Commons Attribution-Share Alike 4.0 International License [8] and (C) NIFS-GM (adapted from Park et al [9] published under Creative Commons Attribution NonCommercial License 4.0 International License [10]). MI-GM: minimally invasive device; NIFS-GM: noninvasive fluid sampling; NIO-GM: noninvasive optical glucose monitoring.

Three main types of noninvasive glucose monitoring devices are currently available: (1) noninvasive optical glucose monitoring (NIO-GM), based on optical glucose monitoring, (2) noninvasive fluid sampling (NIFS-GM), based on fluid sample glucose estimation, and (3) minimally invasive devices (MI-GM), which use a sensor inserted into the subcutaneous tissue [5]. Figure 1 illustrates an example of each type of noninvasive and minimally invasive blood glucose monitoring.

NIO-GM estimates glucose concentration from energy absorption, reflection, or scattering of a light beam directed through the tissue [11]. These devices have the advantage of being both portable and inexpensive. NIO-GM technology includes fluorescence spectroscopy, which may lead to toxicity from fluorophores [12,13]; Raman spectroscopy, criticized for its lengthy spectral acquisition time and poor signal-to-noise ratio [14,15]; photoacoustic spectroscopy, which introduces noise from its sensitivity to environmental factors [15,16]; optical coherence tomography, which is overly sensitive to skin temperature [17]; and occlusion spectroscopy, known to result in signal drift [18]. In contrast, we have developed a NIO-GM device using near-infrared absorption spectroscopy, which is more practical and cost-efficient than those described above [19-23].

### Objectives

**Figure 2:**
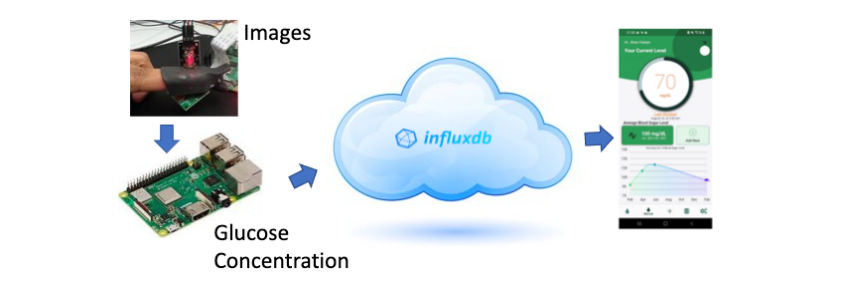
Overview of the proposed noninvasive blood glucose monitoring system.

Here, we describe the development of a novel noninvasive glucose monitoring system that uses the computing power of sensors and Internet of Things devices to continuously analyze blood glucose from a microcomputer and a sensor embedded within a clip positioned on the finger or ear. The prototype uses infrared spectroscopy to create images of the rotational and vibrational transitions of chemical bonds within the glucose molecule, and incident light reflection to measure their corresponding fluctuation. The images are converted into an array list, which is used to provide entries for an artificial neural network (ANN) to create an estimate of blood glucose concentration. The prototype is easy to use and is paired with a mobile app for free-living environments. Figure 2 shows an overview of the proposed system.

## Methods

### Physical Theory

Our prototype detects blood glucose concentration using noninvasive absorption spectroscopy optical glucose monitoring [24]. It is based on the Beer-Lambert law of absorption that is shown in equation 1 [24].



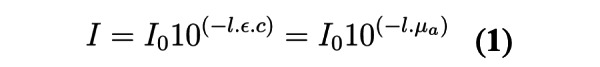



where *I*_0_ is the initial light intensity (*W*/*cm*^2^), *I* is the intensity of the *i*^th^ at any depth within the absorption medium in *W*/*cm*^2^, *l* is the absorption depth within the medium in centimeters, *e* is the molar extinction coefficient in L/(mmol cm), and *c* is the concentration of absorbing molecules in mmol/L. The product of 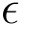 and *c* is proportional to the absorption coefficient (*µ_a_*).

The concentration of absorbing molecules is based on the above equation. However, the effect of other blood components and absorbing tissue components affects the amount of light absorbed. As a result, the total absorption coefficient is the summation of the absorption coefficients of all the absorbing components [25]. Then, to minimize the absorption due to all the other components, the wavelength of the light source should be chosen so that the light source is highly absorbed by glucose and is mostly transparent to blood and tissue components.

### Hardware Configuration

We used Internet of Things technologies to leverage power computing and low energy consumption of sensor devices and a Raspberry Pi camera for building the glucose-monitoring prototype [26]. Although the Raspberry Pi camera captures images, a laser light captures absorption. The specifications of the laser light can be found in Table 1.

**Figure 3:**
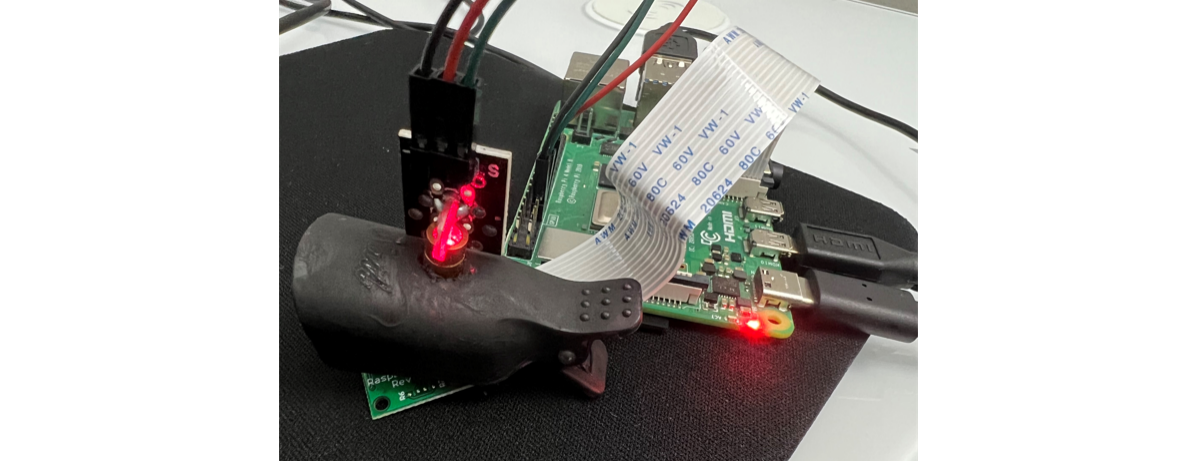
GlucoCheck device.

A small clip that can be positioned on a finger or earlobe holds the laser on the top half and the camera on the bottom. Figure 3 depicts the elements of the prototype (Raspberry Pi, camera, and laser light). The prototype has been named GlucoCheck.

**Figure 4:**
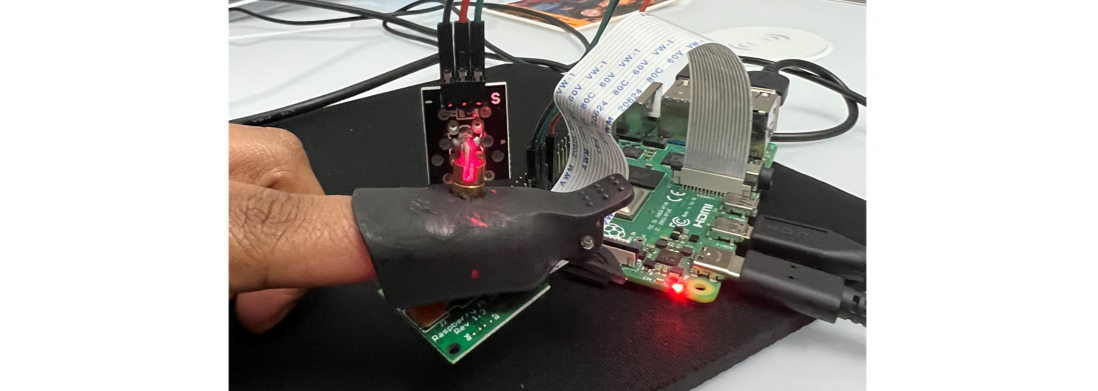
Prototype clipped to the finger.

**Figure 5:**
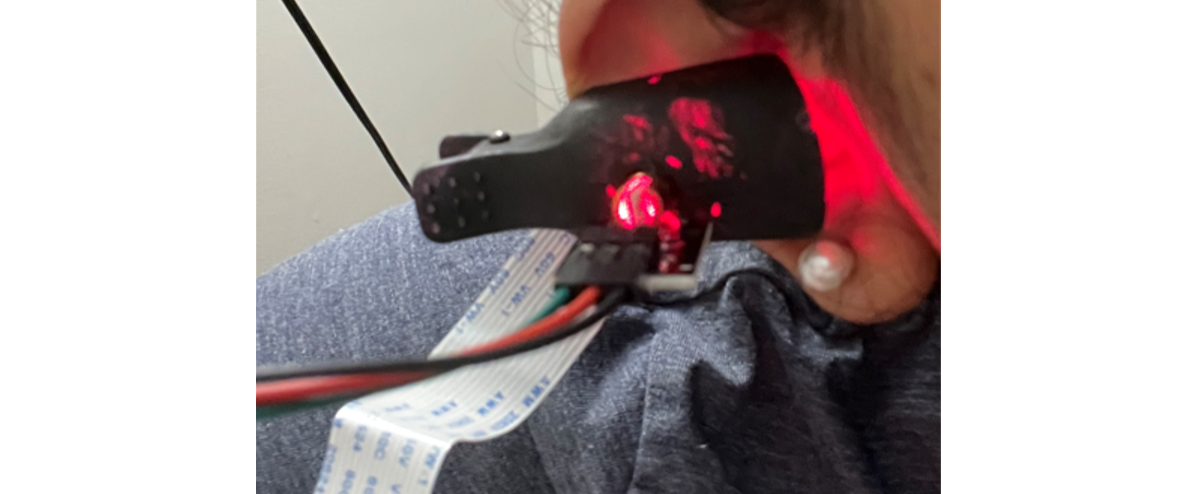
Prototype clipped to the ear.

The Raspberry Pi camera captures one image every 8 seconds over 2 minutes, for a total of 15 images. Brightness and contrast levels are set to 70 cycles/degree, camera ISO sensitivity is set to 800, and resolution is set to 640 × 480. Figures 4 and 5 show the prototype attached to the finger and ear, respectively.

The materials for the GlucoCheck prototype cost approximately US $79-$154 in 2022, depending on the availability of chips, which has been an ongoing issue in recent months. Typically, computer boards are abundant, but 2022 saw a shortage of chips, leading to inflated prices compared to previous years.

**Table 1:** Light laser specifications.

Brand	Icstation
Model number	KY-008 5mW Red Laser Transmitter
Module	Infrared
Part number	276810
Working voltage	5 mW
Wavelength	>650 nm
Size	24 × 15 mm or 0.94 × 0.59 inches (length × width)

### ANN Model

Due to the large number of images used by our prototype, we use a convolutional neural network (CNN/ConvNet) approach. The convolutional layer is the first layer of a CNN network and is the main building block that handles most of the computational work. We imported necessary libraries including Tensor Flow, Keras, MobileNetV2, Matplotlib, and Numpy. The image data set was converted into arrays with preprocessing, then stored in a list format with assigned labels. Finally, the images were appended to a single data array with a corresponding label array and data augmentation techniques were used to train our model, including cropping, zooming, height and width shift, and horizontal flipping.

**Figure 6:**
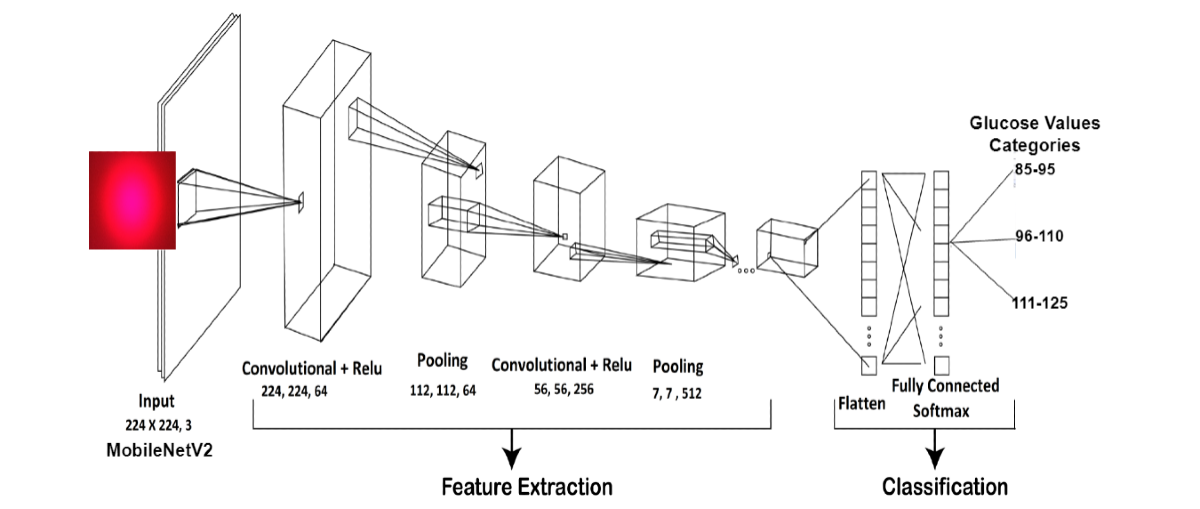
Artificial neural network model used for glucose estimation. ReLu: rectified linear unit.

Our base model, MobileNet-v2, is a lightweight, 53-layer deep CNN method used to improve the classification of images with a limited data set. The next step was to build the head model, which sits on top of the base model. The next layer is the activation layer, which uses the rectified linear unit (ReLu) activation function [27]. The ReLu is a piecewise linear function that will output the input directly if it is positive; otherwise, it will output zero. It has become the default activation function for many types of neural networks because a model that uses it is easier to train and often achieves better performance [28]. The next layer is the pooling layer, which incorporates feature-down sampling. It is applied to each layer in the 3D volume. The fully connected layer, which involves flattening, is the final step. The entire pooling feature map matrix is transformed into a single column, which is then supplied to the neural network for processing. We put these attributes together to make a model using the fully linked layers. Finally, we classified the output using a “Softmax” activation function. The ANN model was trained using the Adam technique, which included a total of 20 epochs, a batch size of 1, and an initial learning rate of 1e-4, and a 0.5 dropout was considered. The next step was to train and test the model; 80% of the data was used for training the model, and 20% was used for testing the model. Figure 6 shows the ANN used for our glucose estimation process.

### Cloud Integration for Real-time Measures

The glucose concentration obtained from the ANN model is sent to the cloud using HTTPS. Next, we configure an InfluxDB [29] database in the cloud to store the data. InfluxDB is an open-source time-series database developed by the company InfluxData. It is written in the Go programming language for storing and retrieving time series data in fields such as operations monitoring, application metrics, Internet of Things sensor data, and real-time analytics. InfluxDB is flexible enough to store data from each subject separately using tags. The integration with the cloud uses the Raspberry Pi, which is connected in real time, and the computed values are displayed on a mobile app for the user.

### Model Testing

**Figure 7:**
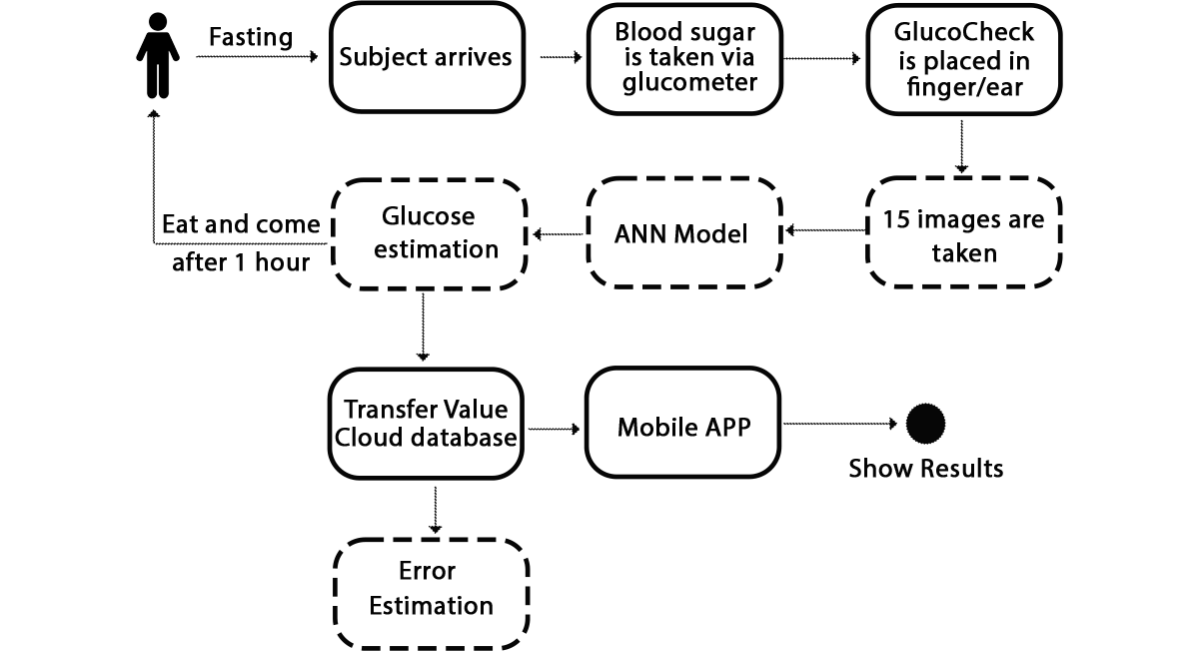
Method workflow.

Glucose data from 8 individuals were used to train and test the model. Each participant was asked to fast for one hour following an unstructured meal prior to the testing visit. Blood glucose concentration was estimated using a commercially available glucometer (FORA 6 Connect BG50 Blood Glucose Starter Testing [17]), according to manufacturer instructions. The GlucoCheck prototype was used to capture images from each participant at two positions: the index finger and the earlobe. As mentioned previously, 80% of the data was used for training the model and 20% of the data was used for testing. The LabelBinarizer module of the Python library sklearn was used to convert the image data to a binary format and store it in an array associated with its corresponding labels/categories (85-95 mg/dL, 96-110 mg/dL, 111-125 mg/dL). Data augmentation (cropping, zooming, height and width shift, horizontal flipping) was used to enlarge our data set for training and testing the model. The data were then passed to our model for glucose estimation. Separate models were developed for images from the finger and images from the earlobe. Figure 7 illustrates the workflow of the protocol.

### Ethical Considerations

For this pilot study, the following ethical considerations were in place. First, the Institutional Review Board of Kennesaw State University approved the study (IRB-FY22-318). In addition, participation in the study was voluntary. Participants were free to opt in or out of the study at any time. Informed consent was required to inform the participant about the study’s purpose, risks, and funding before they agreed or declined to join. Finally, any personally identifiable data were anonymized and kept confidential for the research group.

## Results

### Experimental Data

**Figure 8:**
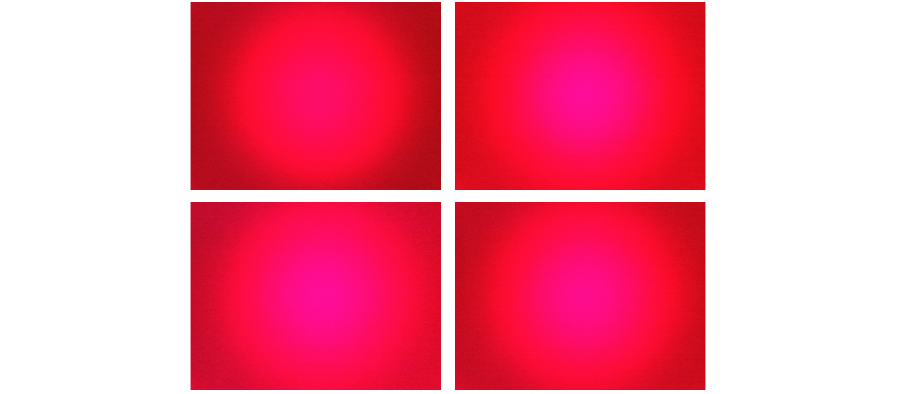
Fingertip images collected from volunteers.

**Figure 9:**
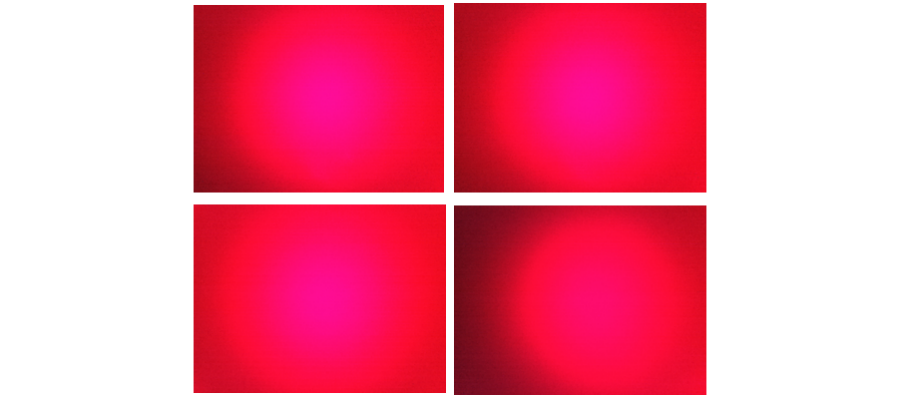
Ear/earlobe images collected from volunteers.

Figure 8 shows images collected from a finger. The images were taken after the finger prick at seconds 8 (top left), 16 (top right), 24 (bottom left), and 32 (bottom right). Figure 9 shows images collected from an earlobe at seconds 8, 16, 24, and 32 after the finger prick.

All the images were then appended to a single data array with a corresponding label array. Then, we performed data augmentation (cropping, zooming, height and width shift, horizontal flipping), which allowed us to expand the variety of data available for training the model as we had a minimal amount of data. The data were then passed to our model for glucose estimation.

### Accuracy Evaluation

The accuracy of the model was assessed with a confusion matrix, which illustrates the proportion of images that were correctly classified. Blood glucose values were grouped as 111-125 mg/dL, 85-95 mg/dL, and 96-110 mg/dL, shown along the x and y axes.

**Figure 10:**
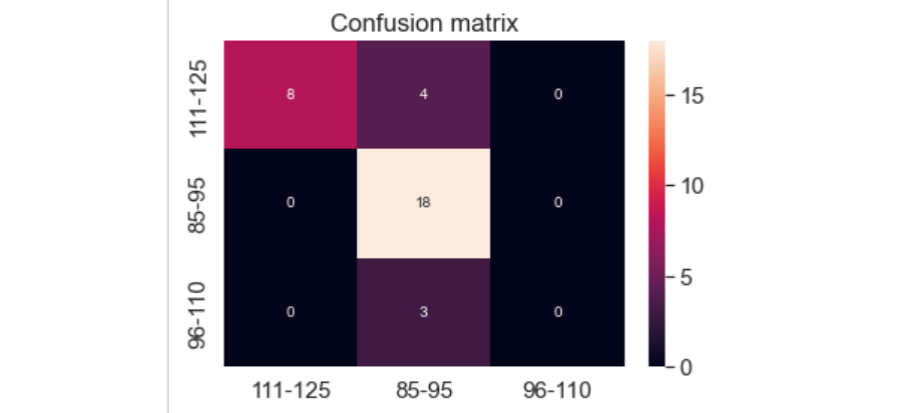
Confusion matrix of finger artificial neural network model. The x-axis refers to the correct estimates, while the y-axis shows incorrect estimates. The unit for all x and y values is mg/dL.

Figure 10 shows the confusion matrix for the glucose estimates when worn on the finger, and indicates a 79% accuracy of the ANN model. The ANN model classified 8 images correctly and 4 images incorrectly in the 111-125 mg/dL category. For the 85-95 mg/dL category, 18 images were correctly classified and 0 images were classified incorrectly. All 3 images in the 96-110 mg/dL category were incorrectly classified. This poor level of accuracy is due to the limited data set for these values.

**Figure 11:**
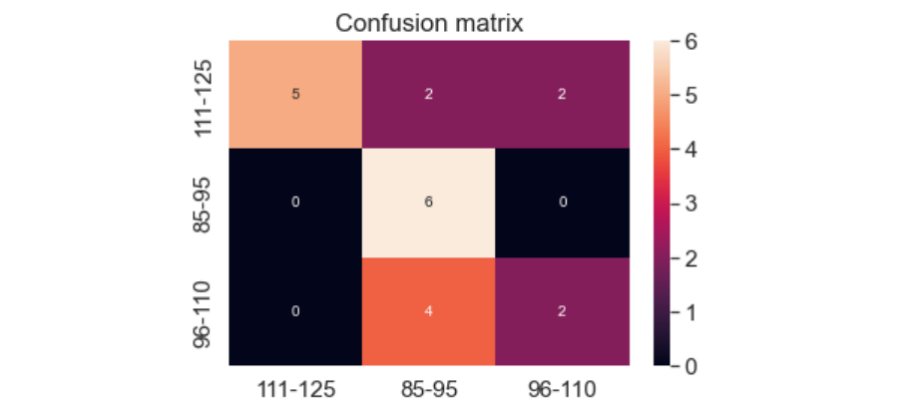
Confusion matrix for ear artificial neural network model. The unit for all x and y values is mg/dL.

Figure 11 shows the results of the ANN model for the ear image data set, which achieved around 62% accuracy. The model classified 5 images correctly and 4 images incorrectly in the 111-125 mg/dL category. In addition, 6 images were correctly classified and 0 images were classified incorrectly in the 85-95 mg/dL category. Finally, 2 images were correctly classified and 4 images were incorrectly classified in the 96-110 mg/dL category.

### Mobile App

**Figure 12:**
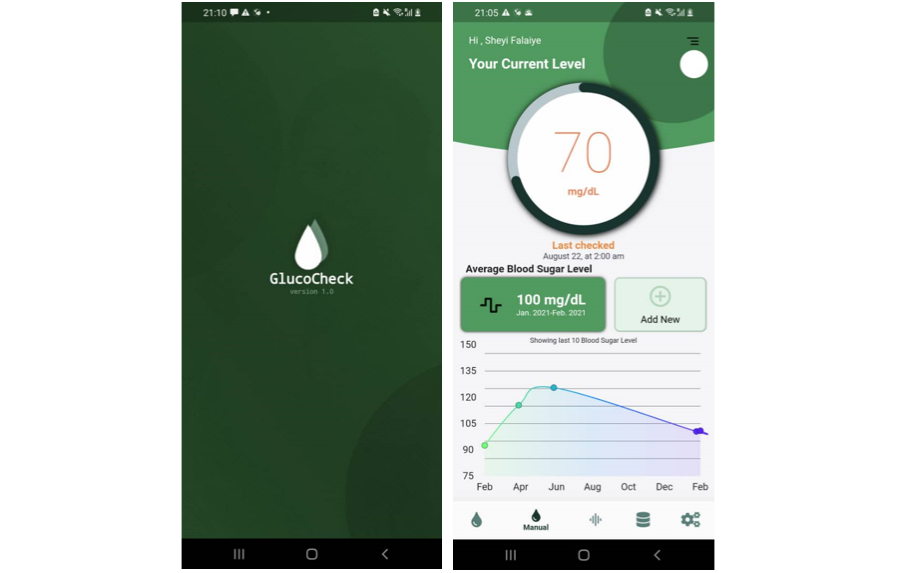
Mobile app interface showing blood glucose level.

Our mobile app “GlucoCheck” is connected to our cloud InfluxDB database and provides continuous glucose monitoring and history data for users. Users can review their current glucose measurement and also view a chart of their previous measurements, allowing them to track glucose variation over a specific period of time. Figure 12 shows the initial screen on the app (left) and the display of glucose readings from the prototype (right).

**Figure 13:**
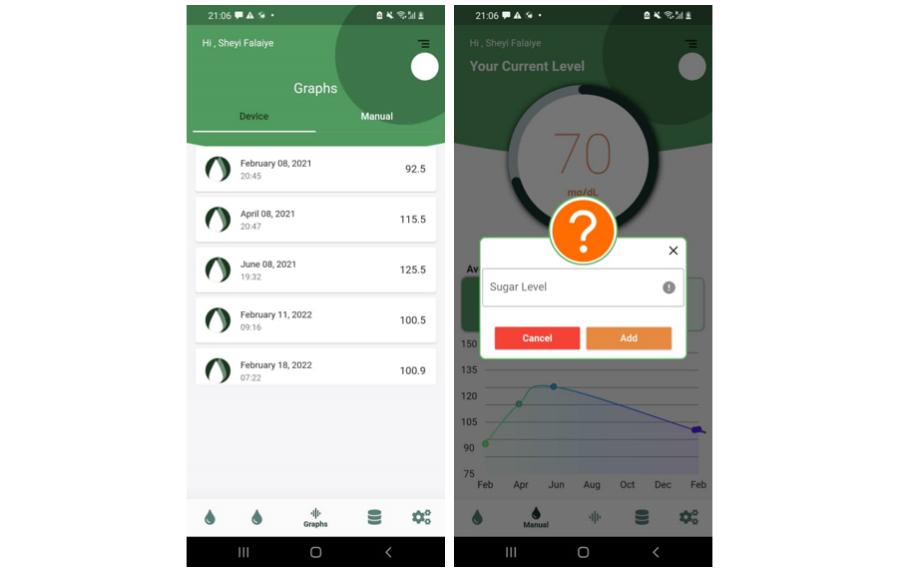
Option to enter glucose level manually. GlucoCheck readings (left) can be compared with other glucometer measures, entered by the user manually (right).

Users may also enter readings from a glucometer into the app to track and compare measurements from other devices, as illustrated in Figure 13.

## Discussion

### Principal Findings

Here we detail and test a novel NIO-GM prototype that relies on an ANN and camera-based technology and is associated with an app that is user-friendly. Results indicate that these optical techniques and machine learning methodologies can effectively measure blood glucose when the light is transmitted and absorptive through the skin tissue. GlucoCheck had an acceptable 79% accuracy when images from fingers were analyzed and 62% accuracy for images from the earlobe position.

Table 2 compares GlucoCheck with previously tested techniques. The potential of GlucoCheck is comparable with other studies, but it has advantages over other technologies. The use of an integrated computer board (Raspberry Pi) and integration with the cloud gives GlucoCheck the unique ability to display values in real time via a mobile app. Additionally, the optional earlobe position of GlucoCheck is unique and allows for the device to be developed as an earring.

**Table 2:** Comparison of this study with previous work.

Study	Body part	Technique	Number of subjects	Accuracy	Real-time	Mobile app	Year
This study (GlucoCheck)	Finger/earlobe	Binary format of image and convolutional neural network	8	79%	Yes	Yes	2022
[19]	Finger	Infrared-multivariate calibration model	3	N/A^a^	No	No	1992
[20]	Finger	Histogram and artificial neural network	514	90%	No	Yes	2019
[21]	Oral mucosa	Attenuated total reflection and hollow fibers	131/414	86.3%	No	No	2018
[22]	Finger and wrist	Reflected optical signal	12	Correlation of 0.86	No	No	2019
[23]	Forearm	Spectra analysis of tissue light path	1	87.5%	No	No	2003

^a^N/A: not applicable.

### Limitations

Future research is needed to address several limitations in the development of a more reliable noninvasive blood glucose prototype based on light. First, a large amount of data is needed for training a machine learning and deep learning model for complicated tasks. Collecting data from people with diabetes is often time-consuming and expensive compared with other tasks. Consequently, many studies face a shortage of data during their research cycles [30-35]. In this preliminary work, we used data augmentation techniques to compute additional data points from our preliminary data set. Additional data will be needed for the ANN model to detect the exact glucose value instead of a range.

Second, depending on the type of radiation used, a viable NIO-GM must account for differences in skin pigmentation, surface roughness, skin thickness, breathing artifacts, blood flow, body movements, and ambient temperature [36]. Accurate measures of the absorption (scattering) properties within human skin remains challenging in biomedical optics and biomedical engineering [37]. Similarly, skin roughness and pigmentation can affect light distribution when propagating through the skin [38]. These factors must be addressed in future technology. Finally, the prototype enclosure design must be comfortable and usable to be effective.

### Conclusion

In this paper, we have presented a noninvasive glucose monitoring system that leverages the computational power of Internet of Things devices and can be used for diabetes management. The prototype is based on images taken from the finger or ear, and does not require blood samples. An ANN model was used to classify and estimate blood glucose concentrations from the images. When using images from the finger, the accuracy of GlucoCheck was 79%. For images taken from the ear, the accuracy was attenuated to 62%. Though the current data set is limited, these results are encouraging. Future studies are needed to address three main limitations: (1) the size of the database (by expanding the data collection process); (2) the prototype enclosure design (by working with biomedical and hardware engineers); and (3) the external factors (by analyzing the impact of skin color, skin thickness, and ambient temperature, among others). If successful, this prototype will be an attractive, life-changing technology for people with diabetes.

## Abbreviations

ANN: artificial neural network
CNN: convolutional neural network
MI-GM: minimally invasive device
NIFS-GM: noninvasive fluid sampling
NIO-GM: noninvasive optical glucose monitoring
ReLu: rectified linear unit
